# Letter to the Editor about the article: Reconstruction of a 3D printed endoprosthesis after joint preservation surgery with intraoperative physeal distraction for childhood malignancies of the distal femur

**DOI:** 10.1186/s13018-024-04541-1

**Published:** 2024-01-18

**Authors:** Mikel San-Julián, Jorge Gómez-Álvarez, José María Lamo-Espinosa

**Affiliations:** Department of Orthopedic Surgery, Clínica Universidad de Navarra, Pamplona, Spain

Dear Editor-in-chief of Journal of Orthopaedic Surgery and Research

We have carefully read the article *Reconstruction of a 3D printed endoprosthesis after joint preservation surgery with intraoperative physeal distraction for childhood malignancies of the distal femur* (Gong et al. Journal of Orthopaedic Surgery and Research (2023) 18:534), and we were surprised that the authors performed intraoperative physeal distraction for metaphyseal osteosarcoma.

Physeal distraction before resection in pediatric bone sarcomas, as described by Dr. J. Cañadell almost 40 years ago, was indeed for achieving a safe margin of resection in pediatric metaphyseal sarcomas near or even in contact with the growth plate. A type I Salter and Harris’s epiphysiolysis is achieved, so that a small layer of growth plate cells covers the metaphyseal edge of resection, while most of the growth plate cells remains together to the epiphysis, inside the patient. Thus, we can preserve the joint, the ligaments attachments and most of the growth potential in many cases. The original technique´s paper was in the reference list of this paper [1, 2].

Having a huge experience in orthopedic oncology as well as in pediatric orthopedics, Cañadell knew from his experimental studies in lambs, that to obtain that, it is necessary a low-speed distraction with external fixator (1 mm/day). Also, he demonstrated that a fast-speed distraction (minutes) the growth plate will broke at the wrong site (as it is seen in Fig. 2 of this article). Cañadell's technique was published to expand its use throughout the world and could help in the treatment of patients with bone sarcomas. However, if modifications are made to the technique, the results are not as expected, as observed in this work.

In the original technique, the distraction is performed in the last days of neoadjuvant chemotherapy, the patients do not have to be hospitalized (they are at home) and the pain they experience when epiphysiolysis occurs is controlled with NSAIDs at home. In this regard, we do not agree with the authors' statement about the need to modify Cañadell´s technique due to the pain of epiphysiolysis. In this regard, the authors cite Betz et al. [3] However, in the original article by Betz et al. they only describe “some discomfort” when epiphysiolysis occurs.

The main problem with high-speed intraoperative distraction is that the main objective of the technique is lost, and it cannot be ensured that a safe resection margin is achieved in tumors close to or in contact with the growth plate. This occurs because, at such speed, a type 1 epiphysiolysis is not achieved, but rather an epiphysiolysis with an uncontrolled fracture line is obtained. The errors that occur if Cañadell's original technique is modified or attempted to “simplify” have previously been published [1, 3–9]. In Cañadell's technique, it is common to observe a macroscopic blue color in the epiphyseal region, indicating most of the epiphysis, while on the tumor side, the color is red. As seen in Fig. 2B, C from the paper, this color pattern is reversed, suggesting that the epiphysiolysis occurred at an incorrect level, not respecting the irregular morphology of the physis at that level. In the worst-case scenario, an uncontrolled fracture at that level could lead to an epiphysiolysis type II pattern (as inferred in the mentioned figure), potentially compromising the distal margin of resection. This is a consequence not only of the distraction rate but also due to the use of Kirschner wires, which do not transmit forces uniformly within the epiphysis.

In our series of 168 patients operated on with the Cañadell technique [10], three patients suffered a fracture in the wrong place. However, in this study this occurred in all 7 patients. Additionally, because the growth cartilage is removed, further growth is not possible in these cases, something that is achieved with the original Cañadell technique in most cases.

Furthermore, in the study we detected that seven patients had the bone tumor at a mean distance from the growth cartilage of 17 mm. In these cases, perhaps a metaphyseal osteotomy would have been sufficient to obtain a safe margin, without the need to perform a physeal distraction. Also, we would like the authors to clarify why they consider that the proximal osteotomy must be performed 3–5 cm from the tumor while in the distal osteotomy 1 cm is considered the minimum margin. Furthermore, we would like to ask why the growth plate is not “used” as a barrier and it is intentionally irregularly disrupted.

King regards,

## Data Availability

No datasets were generated or analysed during the current study.
